# Investigating Eye Movements to Examine Attachment-Related Differences in Facial Emotion Perception and Face Memory

**DOI:** 10.3390/jimaging11020060

**Published:** 2025-02-16

**Authors:** Karolin Török-Suri, Kornél Németh, Máté Baradits, Gábor Csukly

**Affiliations:** 1 Department of Cognitive Science, Faculty of Natural Sciences, Budapest University of Technology and Economics, Műegyetem rkp. 3., H-1111 Budapest, Hungary; 2 Department of Psychiatry and Psychotherapy, Semmelweis University, Balassa u. 6., H-1083 Budapest, Hungary; 3 Feinstein Institutes for Medical Research, Institute of Behavioral Science, Manhasset, NY 11030, USA

**Keywords:** emotional face processing, eye movements, attachment style, state/trait anxiety, linear mixed model

## Abstract

Individual differences in attachment orientations may influence how we process emotionally significant stimuli. As one of the most important sources of emotional information are facial expressions, we examined whether there is an association between adult attachment styles (i.e., scores on the ECR questionnaire, which measures the avoidance and anxiety dimensions of attachment), facial emotion perception and face memory in a neurotypical sample. Trait and state anxiety were also measured as covariates. Eye-tracking was used during the emotion decision task (happy vs. sad faces) and the subsequent facial recognition task; the length of fixations to different face regions was measured as the dependent variable. Linear mixed models suggested that differences during emotion perception may result from longer fixations in individuals with insecure (anxious or avoidant) attachment orientations. This effect was also influenced by individual state and trait anxiety measures. Eye movements during the recognition memory task, however, were not related to either of the attachment dimensions; only trait anxiety had a significant effect on the length of fixations in this condition. The results of our research may contribute to a more accurate understanding of facial emotion perception in the light of attachment styles, and their interaction with anxiety characteristics.

## 1. Introduction

### 1.1. Attachment Theory and Models

Attachment theory is built on the assumption that our early childhood relationships with caregivers affect how we form social bonds throughout our lives, and these experiences can have a powerful and lasting impact on our personality [1]. Bowlby postulated the forming of internal working models from our interactions with others, which lead to a set of “rules” that determine our understanding of self and others [2]. Our primary attachment figures (i.e., parents, caregivers) play the most important role in the development of our working models, but later in life, these might be modified by our friendships or experiences in romantic relationships [3,4,5]. The biological function of the attachment system is to prompt the individual to achieve a sense of security by maintaining proximity to caring and supportive individuals (i.e., attachment figures). The system is activated by signs of danger or detachment from the caregiver, which induce anxiety and trigger proximity-seeking behavior. Once the danger has been eliminated, proximity-seeking becomes unnecessary and the individual may focus on other activities, confident in the belief that the person providing security will continue to be available when needed [6].

The development of the attachment system is highly dependent on the quality of interactions with attachment figures. When relationship partners are available and supportive in times of need, a person can develop a sense of attachment security and positive internal working models of self and others. Negative experiences (rejection or unavailability of the caregiver), on the other hand, may lead to the development of negative models of self and others, as well as secondary attachment strategies [6,7]. Security-based (primary) strategies help individuals manage negative experiences constructively and harness positive emotions for enhanced creativity and problem-solving. In contrast, secondary strategies like deactivation can lead to the suppressing of emotions (both positive and negative), while hyperactivating strategies intensify negative affect, limiting the benefits of positive experiences [8].

Research on the adult attachment system focuses on attachment styles—patterns that determine our expectations, emotions, and social behavior as a function of our past experiences [9]. Early concepts of adult attachment styles were derived from the typology of infant attachment, with distinct categories labeled as secure, avoidant and anxious [10], based on the assumption that these categories have parallels in adult (romantic) relationships [3]. However, in the following years, subsequent studies found that an individual’s attachment style might be better conceptualized as a region in a two-dimensional space of two orthogonal dimensions (i.e., attachment anxiety and avoidance), rather than as a discrete, categorical measure [9,11,12]. In this two-dimensional space, a secure attachment style refers to the region where both avoidance and anxiety are low. A securely attached person is confident in the partner’s love and availability and is comfortable with being close to and depending on the other person. The region in which anxiety (fear of rejection and abandonment) is high, while avoidance is low, corresponds to an anxious attachment style. In turn, the region where avoidance is high (resulting in discomfort with intimacy and interdependence), while anxiety is low, could be termed as an avoidant attachment style [12,13]. According to the two-dimensional model of attachment, an individual’s attachment style can be inferred from completing a self-report questionnaire containing items that tap into traits underlying the two orthogonal dimensions, such as the Experiences in Close Relationships (ECR) questionnaire [12]. Authors of a recent meta-analysis noted, however, that according to Bowlby’s [14] original conceptualization of attachment, these two dimensions might be orthogonal in theory but could be oblique in actual practice; therefore, the independence of anxiety and avoidance is neither a requirement nor a necessary assumption. Results of the meta-analysis indeed yielded significant correlations (average effect size = 0.20) among the anxiety–avoidance dimensions of both the original [12] and the revised [15] version of the questionnaire (ECR and ECR-R, respectively), indicating that the two dimensions underlying attachment orientations are not independent of each other. The authors suggested adapting statistical analyses to account for the shared variance between dimensions [16].

### 1.2. Attachment and Emotion Regulation

Attachment styles have been shown to affect our information processing mechanisms [17], especially for emotionally significant stimuli [18,19]. For example, Sheinbaum et al. have shown in a recent study, that participants perceived their own emotional states and appraised daily life social situations differently, depending on their attachment style. According to their results, anxiously attached individuals reported higher negative emotional states and social distress than secure individuals; however, participants with an avoidant attachment style experienced reduced positive emotional states and a decreased desire to be with others following their social interactions, compared to securely attached individuals [20]. A recent meta-analysis examined whether insecure attachment strategies may lead to an attention bias in the processing of emotional information. They also accounted for multiple potential moderating factors, such as stress, the valence and attachment-related relevance of the information, and the attentional stage of participants. According to their results, people high in attachment avoidance show reduced attention to emotional stimuli regardless of moderating factors, reflecting general deactivating strategies. In contrast, those high in attachment anxiety display increased attention to emotional stimuli, particularly under stressful circumstances and during later attentional stages, indicating more situation-specific hyperactivating strategies. However, under less stressful conditions, and during early stages of processing, individuals high in attachment anxiety respond more like those with high attachment avoidance [21].

Insecure attachment may also play a role in emotion dysregulation and symptoms of social anxiety [22], although some studies found that only attachment anxiety is specifically relevant in these disorders, while the avoidance dimension does not contribute to symptoms of anxiety [23]. Furthermore, both attachment anxiety and avoidance have been previously linked to depressive symptoms [24,25], and according to recent studies, this relationship might also be mediated by the (perceived) inability to use proper emotion regulation strategies [26,27].

### 1.3. Attachment Styles and Facial Emotion Processing

Throughout the course of our lives, we participate in social interactions with thousands of our fellow human beings. Our success in these situations depends on our ability to properly convey our emotional states and to understand the intentions of others. Human faces play a key role in these interactions [28]. Based on Paul Ekman’s work, basic human emotions are universal, biologically rooted, and serve essential adaptive functions. These core emotions—such as happiness, sadness, anger, fear, surprise, and disgust—are expressed through consistent facial expressions and physiological responses across cultures. Ekman’s research highlights their evolutionary role in communication and survival, emphasizing how these emotions help humans navigate complex social and environmental challenges [29,30].

Few studies have shown that our understanding of these social-emotional signals might be influenced by our attachment orientations early on, even at the perceptual level, i.e., the processing of emotional facial expressions [18,31]. In a study by Niedenthal et al., participants viewed movies of faces that changed from expressing specific emotions to a neutral expression gradually; the task was to indicate the point where the original expression was no longer visible. According to their results, attachment orientation influenced the perceptual processing of emotional stimuli, with insecurely attached individuals, especially those fearfully (i.e., high in both attachment anxiety and avoidance) attached, perceiving the offset of facial expressions differently from securely attached individuals, particularly under conditions of distress [18]. Another study focused specifically on the hyperactivating strategies characteristic of anxious attachment: they also implemented a morph movie paradigm, where participants viewed movies of faces transitioning either from emotional to neutral (Study 1) or from neutral to emotional expressions (Studies 2–4). Participants were asked to judge the point at which the emotional expression disappeared or emerged. This approach assessed how attachment-related anxiety influenced participants’ perception and accuracy in identifying changes in emotional expressions. Individuals high in attachment-related anxiety exhibited heightened vigilance to emotional cues, perceiving the onset and offset of emotional expressions earlier than others, but this heightened vigilance seemed to impair their accuracy (unless observation time was controlled as well, leaving participants more time to explore), highlighting the link between attachment anxiety and sensitivity to social-emotional signals [31].

### 1.4. Eye-Tracking in Attentional Processes and Emotion Perception

Eye-tracking has been increasingly used in recent years to study attentional processing and facial emotion perception in both neurotypical people [32,33] and individuals living with psychiatric conditions, such as autism spectrum disorder [34], depression [35,36], or social anxiety [37,38].

A meta-analytic review of eye tracking research on attentional biases in anxiety and depression found that anxious individuals show heightened vigilance for threat during free viewing and difficulty disengaging from threat during visual search tasks. In contrast, depressed individuals exhibit reduced orienting to and maintenance of gaze on positive stimuli, along with increased maintenance of gaze on dysphoric stimuli, but no vigilance for threat during free viewing. These findings highlighted the differences in attentional biases between anxiety and depression while also emphasizing the value of eye-tracking for advancing theoretical models and contributing to research on affective disorders [39]. A recent systematic review also examined eye-tracking patterns in individuals with social anxiety disorder (SAD) during facial emotion recognition tasks. The review of 13 studies found that individuals with SAD exhibit hypervigilance–avoidance behavior, particularly toward negative facial expressions, avoiding prominent facial areas like the eyes. These results provide further support for the relevance of using eye-tracking methods when examining facial emotion perception mechanisms [40].

Borderline personality disorder (BPD) has also been a focus of eye-tracking research in recent years [41,42]. BPD is a complex mental disorder characterized by severe emotion regulation deficits, which can be divided into four subcomponents: sensitivity to emotional signals, heightened negative affect, inadequate regulation strategies, and a tendency to choose maladaptive behaviors [43]. Since people with BPD often show signs of disrupted attachment system functioning, i.e., insecure attachment [44,45] as well as deficits or changes in emotion perception [41,42,46], we inferred that attachment insecurity in BPD may indirectly affect facial emotion processing capabilities through heightened emotion sensitivity, or inadequate emotion regulation mechanisms. Therefore, assuming that these deficits in BPD result from quantitative differences in attachment system functioning (i.e., BPD reflects an “extreme” endpoint of attachment dimensions), we hypothesized that attachment insecurity could be related to altered emotion perception in neurotypicals as well. That is, individual attachment orientations may not only contribute to differences of emotion perception in psychiatric conditions (such as BPD).

Eye-tracking studies have also examined attentional processes during face perception in neurotypical individuals. Schurgin et al., for example, investigated how people focus on distinct regions while judging emotions in static facial stimuli, finding that eye movements varied by emotion, such as focusing on the mouth region for joy and on the eyes for sadness. These patterns align with the most prominent diagnostic regions for recognizing each emotion [32]. Another study examined how people focus on these diagnostic facial regions when viewing dynamic expressions transitioning from neutral to emotional. Eye movement patterns showed similar results, with specific regions attracting visual attention earlier and longer, depending on the emotion (e.g., the eyes for anger and sadness, mouth for happiness), reflecting selective attention to expression-specific regions [33].

According to recent studies, these selective attentional mechanisms found among neurotypicals in facial emotion processing might be influenced by their individual attachment styles as well. A study by Jia et al. examined the impact of adult attachment orientations on preference for infant faces using eye-tracking. The results showed that women with higher attachment avoidance spent less time and had fewer fixations on infant faces, suggesting a reduced attentional bias, which may have implications for understanding interactions between children and mothers with insecure attachment [47]. Another study explored how attachment styles influence oculomotor behavior during visual exploration of positive and negative social interactions. While processing negative images was unaffected, individuals with insecure attachment (high avoidance or anxiety) showed reduced accuracy or delayed attention capture for positive images, indicating a higher threshold for recognizing positive emotions [48].

Eye-tracking can also be combined with other biomarkers, such as event-related potentials (ERP-s), to measure facial emotion processing mechanisms in people with different attachment styles. A recent study examined the emotional processing and deactivation strategies of avoidant individuals compared to secure individuals using eye-tracking and ERP measurements. Avoidant individuals showed impaired processing of facial expressions, with less focus on the eyes of emotional (especially angry) faces. They also exhibited distinct neural responses (more positive P100, less negative N170, and larger P300 amplitudes) compared to secure individuals. These findings highlight the unique face-processing characteristics and deactivating strategies of avoidant individuals, which are crucial for understanding their challenges in social interactions [49].

### 1.5. Eye-Tracking in Face Memory Research

Although less prominent than the research on emotion processing with eye-tracking methods, there are some studies that have investigated face memory with eye-tracking paradigms as well. Previous research has focused on the own-race bias [50], age-related differences in face recognition [51] as well as age effects on emotional memory [52]. Eye-tracking has also been applied to neurodivergent populations, such as autism spectrum disorder (ASD), finding differences in fixation patterns that may contribute to face recognition difficulties observed in ASD [53]. However, no prior study has explicitly examined the connection between attachment styles and face memory in neurotypical samples using eye-tracking.

As highlighted in the literature above, eye-tracking is a versatile tool that allows researchers to examine a range of cognitive processes, including attention [47], learning [54], emotion perception [55], and memory [56]. Researchers employ various metrics, including pupil dilation [50,56], total number of fixations within specific regions [47,52,53], and fixation duration, which is widely recognized as a reliable measure of cognitive processing [52,53,56]. Meghanathan et al. for example examined visual memory load in a visual search task, using fixation duration and pupil size as measures. Their results indicated that fixation duration reflected the number of targets both within and beyond working memory capacity, whereas pupil size only indicated memory load when targets exceeded capacity. Fixation duration increased for targets but not distractors, highlighting its sensitivity to both memory and attentional processing loads [56].

In the present study, we utilized fixation duration data to measure attentional processes during facial emotion perception and subsequent recognition performance.

### 1.6. Study Aim and Contents

The primary aim of this study is to investigate the relationship between adult attachment styles and face processing, with a particular focus on emotion perception and memory. By integrating the two-dimensional model of attachment-related anxiety and avoidance, this research examines how these dimensions influence attention allocation to emotional facial expressions and subsequent memory performance. The study further explores the potential moderating effects of state and trait anxiety on these processes, providing a nuanced perspective on how individual differences shape face perception.

This study makes several novel contributions to the literature. First, it extends prior research on attachment and emotion processing by employing eye-tracking technology, offering precise, fine-grained data on fixation patterns as a measure of attentional processes. Second, it incorporates two subsequent tasks—an emotion decision and a face recognition task—to explore attachment-related mechanisms at both perceptual and memory levels, an underexplored area in prior research. Finally, it bridges gaps in the literature by analyzing fixation duration, a robust indicator of cognitive engagement, while addressing interdependencies between attachment dimensions and anxiety characteristics.

## 2. Materials and Methods

### 2.1. Participants

Participants were recruited from the Budapest University of Technology and Economics and received partial course credit for their participation. Recruitment was conducted via an online form containing the original 36-item version of the Experiences in Close Relationships (ECR) questionnaire [12] and the trait subscale of the State-Trait Anxiety Inventory (STAI-T) questionnaire [57], as well as general socio-demographic questions (gender, age, education, place of residence). A total of 50 neurotypical participants (26 male; age range: 18–36 years, mean age = 24.4, SD = 4.84) were selected. All participants were right-handed, as determined by the Edinburgh Handedness Inventory [58], had no history of psychiatric or neurological disorders, were not prescribed any medication known for influencing cognitive functions, and all had normal or corrected-to-normal vision. Written informed consent was obtained from all participants prior to the experiment. The study was approved by the United Ethical Review Committee for Research in Psychology, Hungary (reference number: 2019-89). The study was carried out in accordance with The Code of Ethics of the World Medical Association (Declaration of Helsinki) for experiments involving humans.

### 2.2. Stimuli, Experimental Design, and Procedure

The experimental procedure comprised several key components, including the administration of psychometric questionnaires, computerized experimental tasks, and eye-tracking data collection.

Facial stimuli used in the experimental task consisted of seven morphing levels representing a continuum from sad to happy expressions. These morphed sequences ensured gradual emotional transitions, allowing for a fine-grained analysis of emotion perception. Participants completed two tasks: an emotion decision task and a face recognition task. During the emotion decision task, they viewed each face and categorized it as either sad or happy. In the recognition task, they were later asked to recall whether they had previously seen a given face. After completing psychometric questionnaires assessing attachment styles and anxiety levels, participants underwent the eye-tracking paradigm, where fixation durations, the primary dependent variable, were recorded to assess attentional processing. Participants’ responses and reaction times were also collected.

Detailed descriptions of each aspect of the experimental procedure are provided below.

#### 2.2.1. Questionnaires

Three psychometric instruments were applied in the study:

*Experiences in Close Relationships (ECR):* This 36-item questionnaire assesses attachment-related anxiety and avoidance using two subscales (18 items each) rated on a 7-point Likert scale (1 = “strongly disagree”, 7 = “strongly agree”). Higher scores on either (or both) subscales indicate greater attachment insecurity [12]. Reliability (measured by Cronbach’s alpha) of the avoidance and anxiety subscales in this study was 0.875 and 0.896, respectively.

State-Trait Anxiety Inventory (STAI): The STAI subscales contain 20-20 statements measuring trait and state anxiety characteristics of an individual [57]. Answers are rated on a 4-point Likert scale (1 = “never”; 4 = “always”). Higher scores reflect increased trait and/or state anxiety. In this study, reliability (measured by Cronbach’s alpha) of the STAI-S and STAI-T subscales was 0.871 and 0.852, respectively.

Beck Depression Inventory (BDI-13): The 13-item version of the BDI is used to screen for depressive symptoms in the general population [59]. It contains 13 clusters of 4 statements ranked from 0 to 3 points, corresponding to increasing depression symptoms. Participants should choose the statement that describes their feelings in the preceding week most accurately from each of the 13 clusters. BDI scores were used to screen for depressive symptoms in our sample: 72% of participants (*n* = 36) scored in the normal range (0–4 points), 18% scored in the range of mild depressive symptoms (*n* = 9; 5–7 points), and 10% (*n* = 5) scored in the moderate symptoms range (8–15 points; highest score = 10). However, since reliability of the BDI-13 in the present study (obtained using Cronbach’s alpha) was 0.552, which is considered questionable or not satisfactory, suggesting insufficient reliability, BDI scores were excluded from subsequent analyses.

#### 2.2.2. Materials

On-site questionnaires (BDI-13, STAI-S), as well as the Edinburgh Handedness Inventory, were administered on a Huawei Mediapad M5 Lite 10′ tablet. Experimental stimuli were displayed using a standard LCD monitor (Acer XF240 H 24′; resolution: 1920 × 1080; refresh rate: 60 Hz) with a viewing distance of 76 cm, maintained using the headrest of the eye-tracking device. Experimental stimuli appeared on a grey background in the center of the screen. SMI HiSpeed 1250 (SensoMotoric Instruments GmbH, Teltow, Germany) and the iViewX program were used to record eye movement, and BeGaze 2.1.152 and MATLAB 2014a (Mathworks, Natick, MA, USA) were used for fixation analysis. Presentation of stimuli was provided by MATLAB2008a (Mathworks, Natick, MA, USA), using Psychtoolbox 3.0.9 [60,61] and custom scripts.

#### 2.2.3. Stimuli and Procedure

**Figure 1:**
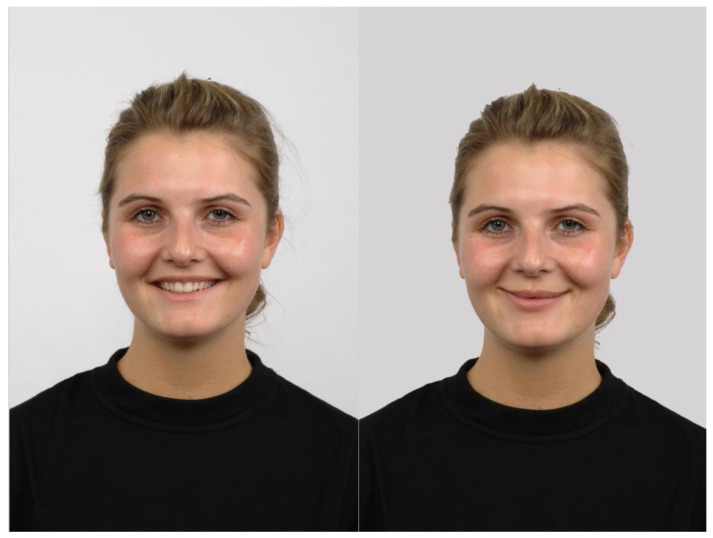
The original (**left**) vs. the edited (**right**) happy face (example; RaFD identity no. 31). Figure 1 has been adapted from the Radboud Faces Database [62]. These images are available on request from the authors’ website (https://rafd.socsci.ru.nl/), no copyright permission is needed.

Facial stimuli were selected from the Radboud Faces Database (RaFD) [62]. A total of 39 adult (19 female and 20 male) Caucasian identities were selected from the frontal view, representing happy, neutral, and sad facial expressions. To standardize happy expressions (since teeth were visible in the original happy faces, which would have led to distorted results on subsequent morphing), Adobe Photoshop CS3 Extended (v10.0) was used to modify neutral faces into smiles while preserving the structural elements of a happy expression (e.g., rounded face, “smiling” eyes; see Figure 1).

**Figure 2:**
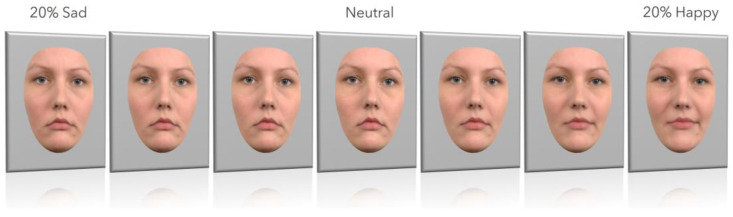
The seven levels of emotional expressions used in the emotion decision task. Outer parts of the facial stimuli (hair, ears, neck) were covered with a grey mask. (Example: RaFD Identity no. 8). Figure 2 has been adapted from the Radboud Faces Database [62]. These images are available on request from the authors’ website (https://rafd.socsci.ru.nl/), no copyright permission is needed.

Morphed sequences of sad-to-neutral, and neutral-to-happy transitions were generated using WinMorph 3.01. Stimuli were adjusted to avoid ceiling effects by limiting endpoints to 80% neutral-20% emotional content. Final stimuli included 7 equidistant levels per RaFD identity, selected from the morphed series of emotional expressions, with outer features (hair, ears, neck) masked in grey. Images were resized to 535 × 700 pixels (10.79 × 14.35 visual angle; see Figure 2).

Participants underwent a 13-point calibration procedure for the eye-tracking device. Upon successful calibration, the experimental instructions were displayed, and participants initiated the task via a button press.

#### 2.2.4. Task 1—Emotion Decision

During the emotion decision task, a face appeared in the center of the screen, and participants had to indicate whether each face was happy or sad by pressing a corresponding key. A total of 19 RaFD identities were randomly selected for each participant, and the 7 emotional levels per identity were presented (19 × 7 = 133 trials). Task 1 was divided into blocks of 19 trials, between which the subject could take short breaks and resume the task by pressing a button. Target stimuli appeared on the screen for 500 ms, followed by a grey mask image for at least 500 ms (response time limit: 2000 ms).

#### 2.2.5. Task 2—Face Recognition

During the face recognition task, in addition to the 19 previously seen identities (old condition), 20 novel identities appeared (new condition), and participants had to indicate whether they had seen the identity before. The order of already seen and new identities was randomized; all identities appeared only once, with a neutral facial expression (39 trials). Stimuli appeared on the screen for 1000 ms, followed by a grey mask image for at least 300 ms (response time limit: 4000 ms).

#### 2.2.6. Eye-Tracking

**Figure 3:**
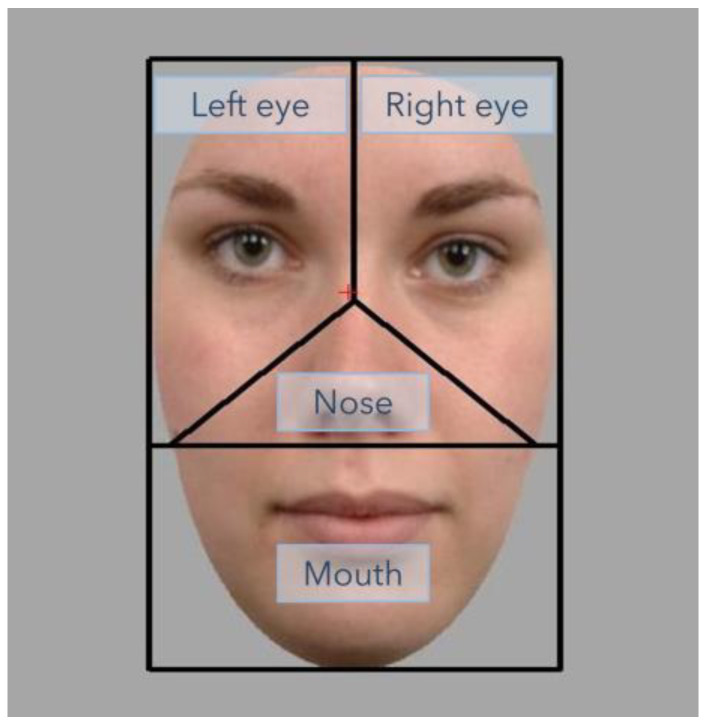
AoI-s based on the Voronoi-method [63]: left eye, right eye, nose, mouth. The two eye regions are named according to the “director’s view” (i.e., anatomically reversed). The position of the fixation cross is marked in red. Figure 3 has been adapted from the Radboud Faces Database [62]. These images are available on request from the authors’ website (https://rafd.socsci.ru.nl/), no copyright permission is needed.

Eye movements of participants were recorded during both tasks. Since there is no clear consensus on the construction of Areas of Interest (AoI-s) in face perception literature, we determined the AoI-s based on the Voronoi-method suggested in an article by Hessels et al. [63]. 4 AoI-s were selected: the left eye, right eye, nose, and mouth regions (see Figure 3). Whether a particular fixation fell into one of the AoI-s was determined by a custom MATLAB script analyzing all fixations initiated during stimulus visibility, with fixation durations expressed as percentages of total visibility time for each AoI (MATLAB 2014a; Mathworks, Natick, MA, USA).

### 2.3. Data Analysis

#### 2.3.1. Data Preparation

Despite the use of a headrest, minor head movements caused distortions in eye movement data. To correct this, the 85% trimmed average of recorded coordinates from the 100 ms pre-stimulus period was calculated. As participants were asked to view a fixation cross before each trial, all in-trial fixation coordinates were adjusted by the difference between this trimmed average and the fixation cross coordinates (X = 960, Y = 440; see Figure 3).

Only fixations initiated during stimulus visibility were considered for data analysis: this meant a 500 ms interval for the emotion recognition task and 1000 ms for the recognition task. The percent of fixations that fell on a given AoI during the visibility of the image were calculated (e.g., a 422 ms long fixation to the nose AoI during a 500 ms emotion recognition trial meant 84.4% for the nose and 0% for other AoI-s).

During the emotion decision task, where stimulus visibility was shorter (500 ms), only the first 3 fixations (initiated during stimulus visibility) per trial were analyzed, while in the face recognition task (1000 ms stimulus visibility), where participants had more time to view the faces, the first 5 fixations were analyzed.

#### 2.3.2. Statistical Analysis

Data analysis was conducted using SAS 9.4 software, with the MIXED procedure (SAS Institute Inc., 2016, Cary, NC, USA), which applies a comprehensive model that allows for both fixed and random effects to be incorporated and analyzed at multiple levels of hierarchically structured data. Separate linear mixed models were created for the emotion decision and face recognition tasks.

Fixation duration served as the dependent variable; stimulus type (happy/sad/neutral face for the emotion decision task; old/new face for the recognition task), AoI-s (left eye, right eye, nose, mouth), and fixation rank (1–3. in the emotion decision task; 1–5. in the recognition task) were used as hierarchical classification variables (clusters); while behavioral measures (reaction times, accuracy), age, and questionnaire scores (STAI-T, STAI-S, ECR avoidance, ECR anxiety) were included as “fixed effect” covariates in both models. Post hoc comparisons among each level of the hierarchical data were performed using Differences of Least Squares Means (LSMEANS) with Tukey’s HSD corrections.

Based on previous literature, the attachment anxiety and avoidance dimensions may not be entirely independent [16]. To investigate this, we checked for collinearity between these two variables to determine if there was a significant linear relationship between them. Perfect collinearity occurs when there is an exact linear relationship between two variables, with a correlation coefficient of either 1 or −1. Strong collinearity between explanatory variables can violate the assumptions of a multivariate linear model. In our sample, both Pearson’s correlation coefficient (r = 0.081, *p* = 0.577) and Spearman’s rank correlation coefficient (ρ = 0.080, *p* = 0.580) indicated no significant collinearity between the attachment anxiety and avoidance subscales of the ECR questionnaire.

## 3. Results

### 3.1. Demographics and Basic Descriptive Characteristics

Table 1 summarizes the demographic and questionnaire-based characteristics of the sample. Percentiles (25th, 50th, 75th) were used to determine reference values for the dependent variable (fixation length) estimates in LSmeans analyses.

### 3.2. Model 1—Fixation Duration in the Emotion Decision Task

Model 1 assessed fixation duration within a three-level hierarchical structure: fixation rank (1st–3rd) nested within face regions (AoI-s: left eye, right eye, nose, mouth), which were nested within stimulus type (sad, happy, neutral). Fixation duration was the dependent variable, with accuracy, reaction time (RT), age, and questionnaire scores (ECR avoidance, ECR anxiety, STAI-T, STAI-S) as fixed effects.

Key findings of the Model suggest that the type of emotion influences fixation durations: fixations were longest for sad faces, shorter for happy faces, and shortest for neutral faces (all *p* < 0.0001). Anxiety and attachment orientations also predicted fixation duration: trait (STAI-T) and state anxiety (STAI-S) were strong predictors of the length of fixations (*p* < 0.01), while attachment anxiety (ECR Anxiety) also significantly predicted fixation duration (*p* = 0.035), whereas attachment avoidance showed a weaker effect (*p* = 0.078). Face Regions (*AoI-s*) also influenced fixation durations, which were significantly longer on the left eye compared to the right eye, nose, and mouth (all *p* < 0.01).

Results for random effects, fixed effects, and interaction outcomes of the model are summarized in Table 2. Detailed results of the model are available in the online supplementary material (Section S1).

**Highlights of Model 1 (see Table 2):**

- **Random Effects:** Fixation rank and AoI-s significantly explained variance in fixation duration (3.2% and 4.8%, respectively), while stimulus type approached significance (explaining 12.1% of variance).
- **Fixed Effects 1:** Significant predictors of fixation duration included STAI-T, STAI-S and ECR avoidance, with ECR anxiety showing a tendency toward significance.
- **Fixed Effects 2 (updated model):** Removing non-significant fixed effects improved the model fit. STAI-T and STAI-S scores remained strongly significant, while ECR anxiety also showed significant effect, but ECR avoidance only showed tendency.
- **Interactions (from the updated model):** Stimulus type, AoI-s, and fixation rank had significant main effects on fixation duration. Interactions between the predictors revealed that fixation durations differed significantly at different levels of the hierarchical model.

Post hoc analysis of differences of least squares means revealed significant differences between all three types of emotional expressions (i.e., stimulus type): fixations were longest for sad faces (t = 30.82, *p* < 0.0001), shorter for happy (t = 18.33, *p* < 0.0001), and shortest for neutral faces (t = 7.67, *p* < 0.0001). Furthermore, fixation lengths differed significantly between sad and happy (t = 8.84, *p* < 0.0001), sad and neutral (t = 16.37, *p* < 0.0001), and happy and neutral faces as well (t = 7.53, *p* < 0.0001). At the level of AoI-s, only the left eye region differed significantly from all other areas, with longer fixations to the left eye than all other areas (right eye: t = 3.43, *p* < 0.01; nose: t = 4.11, *p* < 0.001; mouth: t = 5.82, *p* < 0.0001). Fixation ranks (1–3.) did not differ significantly from each other (1–2: *p* = 0.99; 1–3: *p* = 0.084; 2–3: *p* = 0.074). Given the lack of differences between fixation ranks, these were not included in any further analyses.

Differences of LSmeans of the *stimulus type × AoI* interaction were further analyzed in the context of fixed effect covariates (ECR avoidance, ECR anxiety, STAI-T, and STAI-S scores). As seen in Table 1, percentile scores were calculated for all questionnaires to determine reference values for the calculated estimates of the dependent variable. The model was used to estimate fixation duration according to scores (25th, 50th and 75th percentile) on a given questionnaire, by calculating a linear estimate based on actual values of participants (considering the covariates as well). Detailed results of LSmeans analyses can be found in the online Supplementary Materials (Sections S2–S5).

**Figure 4:**
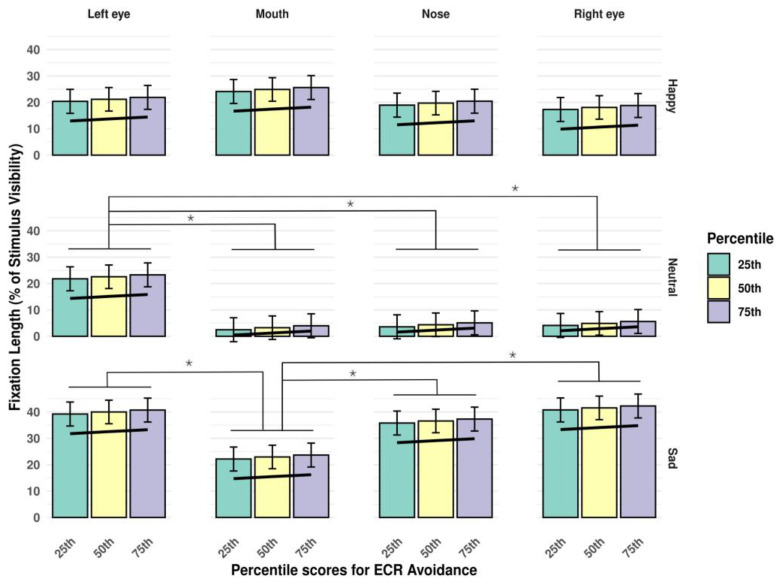
Interaction of *stimulus type* and *AoI*, in the context of percentile scores on the ECR avoidance subscale. The trendline highlights the positive association between fixation duration and scores on the avoidance dimension of the ECR. Significant differences between estimated fixation lengths to different AoI-s are marked with an asterisk (*p* < 0.05). Error bars represent standard errors (SE-s).

**Figure 5:**
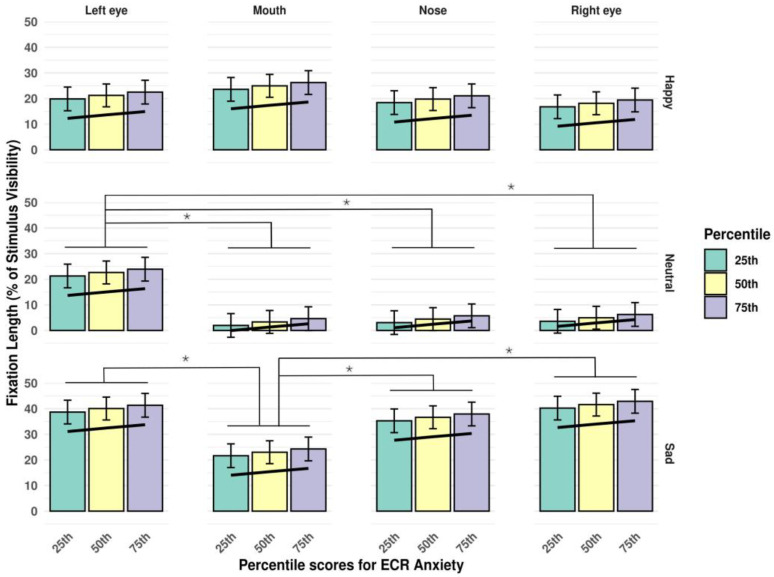
Interaction of *stimulus type* and *AoI*, in the context of percentile scores on the ECR anxiety subscale. The trendline highlights the positive association between fixation duration and scores on the anxiety dimension of the ECR. Significant differences between estimated fixation lengths to different AoI-s are marked with an asterisk (*p* < 0.05). Error bars represent standard errors (SE-s).

The results altogether suggested specific patterns of fixation duration for the different questionnaire measures: we found a positive association between fixation duration and scores on both the avoidance and anxiety dimensions of the ECR (see Figure 4 and Figure 5, respectively).

**Figure 6:**
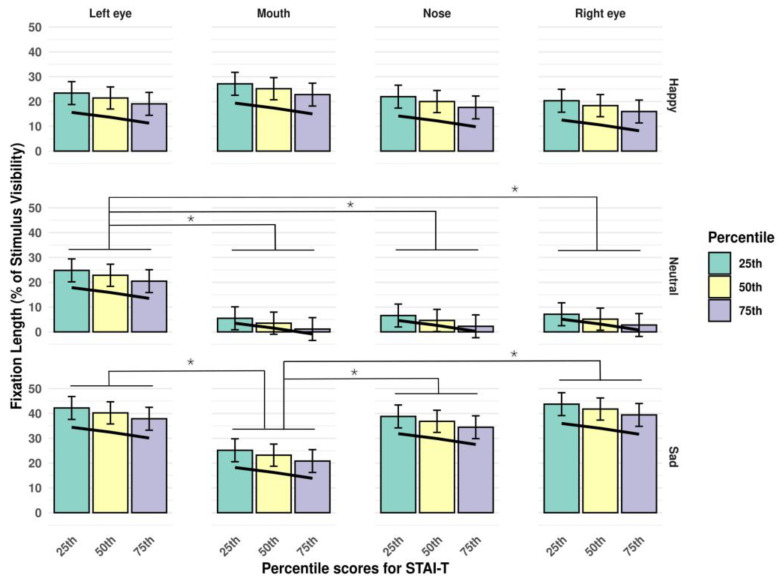
Interaction of *stimulus type* and *AoI*, in the context of percentile scores on the STAI-T subscale. The trendline highlights the negative association between fixation duration and scores on the trait version of the STAI. Significant differences between estimated fixation lengths to different AoI-s are marked with an asterisk (*p* < 0.05). Error bars represent standard errors (SE-s).

**Figure 7:**
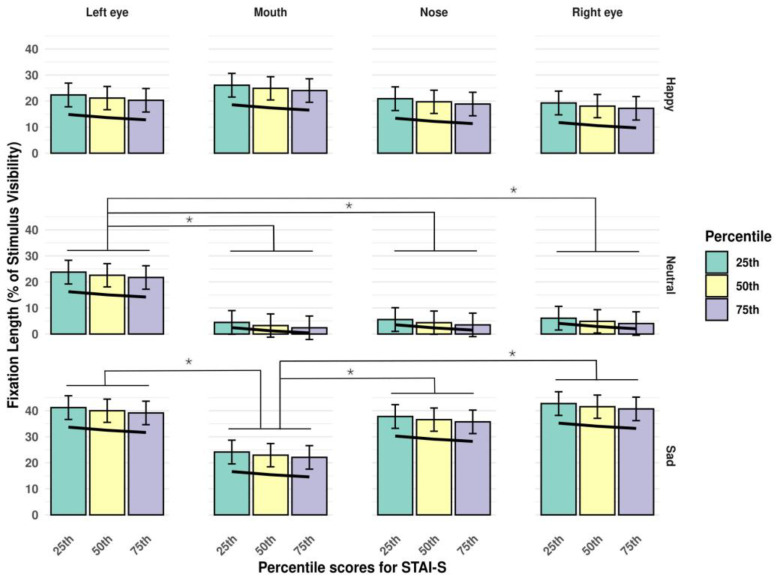
Interaction of *stimulus type* and *AoI*, in the context of percentile scores on the STAI-S subscale. The trendline highlights the negative association between fixation duration and scores on the state version of the STAI. Significant differences between estimated fixation lengths to different AoI-s are marked with an asterisk (*p* < 0.05). Error bars represent standard errors (SE-s).

The opposite effect was observed for anxiety characteristics: both trait anxiety (Figure 6) and state anxiety (Figure 7) showed a negative relationship with the length of fixations.

Furthermore, regardless of the effect of the questionnaires, differential effects of emotion on the length of fixations to different face regions were observed: for sad faces, longest fixations fell on the right eye, followed by the left eye, nose and mouth. For happy faces, fixations on the mouth were longest; the left eye area was second, followed by the nose, and the right eye received the least attention. When viewing neutral faces, fixations were longest for the left eye, followed by the right eye, nose and mouth.

### 3.3. Model 2—Fixation Duration in the Face Recognition Task

Model 2, structured similarly to Model 1, examined fixation duration across fixation rank (1st–5th) nested within face regions (AoI-s: left eye, right eye, nose, mouth), and stimulus type (old vs. new faces). Fixation duration was the dependent variable in the model, while all other variables (accuracy, RT, age, questionnaire scores) were given as fixed effects.

Key findings of the Model revealed that trait anxiety (STAI-T scores) was the only significant predictor of fixation duration (*p* = 0.02), suggesting that individuals with higher trait anxiety exhibited shorter fixations overall. Different face regions (*AoI-s*) also influenced the length of fixations: each facial region (left eye, right eye, nose, mouth) differed significantly in fixation duration (all *p* < 0.0001) during the recognition task. Fixation duration also varied across fixation ranks (*1st–5th)*, with significant differences at select levels (*p* < 0.05). However, there was no significant difference in fixation duration between old and new faces (*p* = 0.27), indicating that memory-related processes did not strongly influence viewing behavior. Detailed results of Model 2 are available below.

Based on the covariance parameter estimates of the model, the random intercept of fixation rank (*p* = 0.012), significantly explained 6.2% of the variance in fixation duration, while AoI-s (*p* = 0.06) showed a tendency and explained 11.9% of variance, after accounting for the variance explained by fixation rank. Covariance parameters could not be estimated for Level 3 (stimulus type) according to this model.

Analysis of fixed effects revealed that only scores on the STAI-T (F = 6.15, *p* = 0.013) correlated with an estimate of fixation duration that differed significantly from 0. All other fixed effects (age: *p* = 0.07; old RT: *p* = 0.49; old accuracy: *p* = 0.72; new RT: *p* = 0.07; new accuracy: *p* = 0.31; STAI-S: *p* = 0.19, ECR anxiety: *p* = 0.08; ECR avoidance: *p* = 0.27) did not show significant results; therefore, these variables were not included in subsequent analyses.

After removing the non-significant fixed effects, the model was updated to incorporate interactions of classification variables (fixation rank, AoI-s, and stimulus type), as well as calculating LSmeans as post hoc comparisons between each level of the clustered data at different levels of fixed effects (i.e., questionnaire scores). The updated model revealed that AoI-s (F = 106.9, *p* < 0.0001) and fixation rank (F = 3.90, *p* < 0.01) had significant main effects on fixation duration, and their interaction was also significant (AoI × fixation rank: F = 7.74, *p* < 0.0001). However, stimulus type did not have a significant effect (*p* = 0.27), and interactions involving stimulus type were also non-significant (stimulus type × AoI: *p* = 0.39; stimulus type × fixation rank: *p* = 0.77; stimulus type × AoI × fixation rank: *p* = 0.66). Removal of all non-significant covariates resulted in different estimates for the remaining fixed effect as well: scores on the STAI-T (F = 5.22, *p* = 0.02) remained significant, but showed a weaker effect.

Post hoc analysis of differences of least squares means revealed that at the level of AoI-s, every area (left eye, right eye, nose, mouth) differed significantly from all other areas (all *p*-s < 0.0001), while fixation ranks (1st–5th) only differed significantly at two levels of comparison (1st vs. 4th: *p* = 0.015; 2nd vs. 4th: *p* = 0.011). Given the lack of main effect, or any significant interactions involving stimulus type, this was not included in any further analyses.

Differences in LSmeans of the AoI × fixation rank interaction were further analyzed in the context of the only significant fixed effect covariate, STAI-T scores. Detailed results of LSmeans analyses can be found in the online supplementary material (Section S6).

The results of the AoI × fixation rank interaction suggested a specific pattern of fixations related to trait anxiety: we found a negative association between overall fixation duration and scores on the STAI-T. Furthermore, differential relationships between specific face areas and the order of fixations were observed; order of fixations on the left eye: 1st > 4th > 2nd > 5th > 3rd; on the right eye: 3rd > 4th > 5th > 2nd > 1st; on the nose: 4th > 3rd > 2nd > 5th > 1st; and on the mouth: 5th > 4th > 3rd > 2nd > 1st. It is important to note however, that that the difference between the 1st and 2nd fixations to the mouth were not significant at any level of questionnaire scores.

## 4. Discussion

This study investigated the interplay between adult attachment orientations, anxiety traits, and visual attention mechanisms during facial emotion perception and face recognition tasks. By utilizing eye-tracking and implementing two separate linear mixed models for the two experimental tasks, we sought to uncover how fixation length patterns differ as a function of individual differences in attachment and anxiety.

A primary finding from the emotion perception task was that fixation durations were longest for sad faces, followed by happy faces, and shortest for neutral expressions. Eye regions attracted the most attention across all expressions, suggesting that longer fixations on sad faces were driven by increased attention to the eyes. Attachment orientations also influenced fixation patterns: individuals with higher attachment anxiety and avoidance exhibited longer fixations on emotional faces, suggesting heightened vigilance or difficulty disengaging from emotional stimuli. Notably, while correlations showed no significant collinearity between the two attachment dimensions, their influence on fixation patterns suggests interdependence. Anxiety traits also played a role: higher trait and state anxiety correlated with shorter fixations on emotional faces.

Unlike the emotion perception task, fixation durations did not differ between previously seen and new faces, indicating that familiarity did not influence attentional allocation. Furthermore, attachment dimensions did not significantly affect fixation duration during face recognition; trait anxiety was the only significant predictor of fixation lengths, with higher trait anxiety associated with shorter fixations, suggesting that trait anxiety may influence visual attention during recognition tasks, independent of behavioral accuracy.

These findings underscore the influence of attachment and anxiety on visual attention during emotion perception, demonstrating that insecure attachment styles and anxiety characteristics shape attentional engagement with emotional stimuli. Our results support fixation duration as a valuable measure of socio-emotional processing, particularly in relation to attachment differences. While contributing to the understanding of these factors, the study also highlights important limitations and directions for future research.

### 4.1. Model 1—Emotion Decision Task

In the first model, we observed significant variance contributions by face regions (AoI-s) as well as emotional expressions: the estimated average length of fixations were longest for sad faces, followed by happy faces, while neutral expressions received the least attention (i.e., shortest fixations). The analysis of AoI-s showed that the eye regions received the most attention (regardless of emotional expression), therefore it could be inferred that longer general fixations for eyes were an indirect result of people viewing sad faces the longest. This pattern is supported by the interaction between face regions and emotion: according to this, people looked mostly at the eyes when processing sad faces, however, they fixated more on the mouth region of happy faces. This is also in line with previous research that found specific viewing patterns for different emotions comparable to our results, that is, selective attention was observed for eyes in sad faces, and for the mouth region when viewing happy faces [32,33]. However, when interpreting these results, it is important to mention that the variance contribution of random effects in this model was limited, explaining only a small portion of the variance in fixation duration.

Based on previous research, we hypothesized that attachment insecurity would be related to altered emotion perception processes in neurotypical individuals. Earlier studies investigated this assumption by analyzing behavioral indices (e.g., vigilance to social cues) of attachment-related anxiety and avoidance: in a study by Fraley et al. [31], for example, participants were shown movies of faces in which an emotional expression changed to a neutral one, or vice versa. Participants had to indicate the point at which the emotional expression had disappeared or emerged from the face. Individuals showing higher attachment anxiety perceived the offset, as well as the onset, of emotional expressions earlier than others, indicating that individual differences in attachment anxiety (but not attachment-related avoidance) reflect altered vigilance to cues of socio-emotional significance. To our knowledge, few studies have approached the relationship between attachment insecurity and facial emotion perception via eye-tracking paradigms: Silva et al. [48] for example have shown that higher attachment avoidance is related to reduced accuracy for recognizing positive emotions (suggesting a higher processing threshold for such stimuli), while people with higher attachment anxiety showed a delay in fixations to pictures with positive emotional valence, which indicates altered attentional processes regarding positive emotions. In another study, eye movements were analyzed when looking at pairs of adult and infant faces depicting different emotional expressions. Their results suggested that adult attachment significantly modulated visual attentional bias to infant and adult faces, indicating that women with higher attachment avoidance show less attentional bias for infant compared to adult faces, based on the length and number of fixations to facial stimuli [47].

In this study, we also found evidence that attachment orientations affect how we process information from faces even at a basic perceptual level (i.e., the length of fixations to different face regions). Our results indicated that higher levels of both attachment dimensions are associated with longer viewing times for emotional faces, regardless of the depicted emotion (compared to neutral expressions). This finding aligns with prior research suggesting that individuals with insecure attachment styles display altered vigilance to emotionally salient stimuli. For instance, individuals high in attachment-related anxiety exhibit heightened vigilance to emotional cues, reflecting hyperactivating strategies, while those high in avoidance show tendencies to suppress engagement with emotional stimuli through deactivating strategies [21,31]. Furthermore, our data suggested that the anxiety and avoidance dimensions are indeed not independent of each other. Even though collinearity between the two subscales was found to be non-significant (measured by Pearson and Spearman correlation coefficients), the inter-dependency of the attachment dimensions was suggested by the changes observed in both dimensions when other parameters of the model for the emotion perception task have been changed, as well as the analogous association of both dimensions with fixation lengths, which further points to the inter-related nature of the two dimensions [16].

Our results also indicated that fixation patterns during facial emotion perception are modified by individual levels of anxiety: we found that higher levels of both state and trait anxiety (but especially the latter) correlated with shorter fixations for emotional faces. The possible influence of anxiety characteristics on facial emotion perception is still a strongly debated topic: some studies have found specific effects of trait, but not state anxiety on fear-detection performance [64], while others only explored the effect of high trait anxiety on emotion perception, yielding similar results, i.e., participants with higher trait anxiety recognized fearful faces significantly better while they did not differ in recognition of other facial expressions [65]. Contrary to these findings, Cooper et al. found no anxiety-related differences in emotion perception across the six basic emotions (anger, disgust, fear, happiness, sadness, surprise + neutral), suggesting that trait anxiety does not influence the perception of fear (or other emotions) at a basic perceptual level, as has been previously proposed [66]. In the present study, only two emotions (sadness and happiness) were assessed, furthermore, we did not find any effect of behavioral results (neither performance, nor reaction times provided significant estimates on fixation duration), therefore our results are not directly comparable to the above-mentioned studies, but still add to the growing body of evidence that anxiety characteristics might affect facial emotion perception processes.

### 4.2. Model 2—Face Recognition Task

Turning to the results of Model 2, we observed no significant effect of stimulus type on the length of fixations, meaning that participants spent an equal amount of time looking at previously seen and new faces as well. A previous eye-tracking study found a decrease in fixations for repeatedly seen faces, which suggests more efficient scanning of familiar faces, especially after multiple presentations [67]. However, that experiment was conducted over 4 consecutive days—here, participants’ memory performance was assessed immediately after the presentation of stimuli—time constraints providing a possible explanation for the lack of difference observed between fixations for previously seen and new faces.

In contrast to the emotion perception task, we had no specific hypotheses about the relationship between memory for faces and attachment dimensions. Nevertheless, both ECR anxiety and ECR avoidance scores were added to the initial model as covariates, but according to our results, fixation durations during the face recognition task were not significantly influenced by attachment dimensions. We found very little previous research on this topic: one study, where participants also performed a study-test task for emotional and neutral faces found differential brain activity for highly avoidant individuals during the encoding and recognition of emotional vs. neutral faces, while there was no difference in behavioral performance between less and more avoidant participants. However, these conclusions were achieved by measuring ERP correlates of face perception and memory processes, furthermore, the study only focused on differences among the avoidance dimension of attachment, while attachment anxiety was not examined; therefore, the results are not directly comparable to our measurements [68].

The lack of significant results in Model 2 may stem from the task’s constrained design, with fewer trials and a focus on static, neutral facial expressions. The only significant effect we found during the recognition memory task was of trait anxiety—according to the estimations of our model, people with higher trait anxiety tend to look less (i.e., have shorter fixations) to faces when deciding whether they have previously seen a given face. Previous research mostly focused on behavioral indices of the relationship between social anxiety and face memory, with findings suggesting weakened recognition of previously seen happy faces by people with higher levels of social anxiety [69], as well as poorer face identity recognition correlating with increased social anxiety, but not with trait anxiety [70]. Some studies, however, found no associations between either trait or social anxiety and face processing ability [71], while others found evidence of enhanced memory for faces that made fair offers during an economic decision task (compared to unfair proposers); however, higher trait anxiety was associated with reduced fair-related memory for faces [72]. Even though in our study, shorter fixations of people with higher trait anxiety were not related to behavioral results (since neither performance nor reaction times provided significant estimates on fixation duration), the possible modifying effect of trait anxiety in eye movements during memory decisions is still an interesting and novel finding.

### 4.3. Limitations and Future Directions

While this study offers novel insights, several limitations warrant discussion. First, the restricted sample of young, neurotypical adults limits the generalizability of our findings to broader or clinical populations [34,35,36,41]. Future studies should incorporate diverse demographic groups and explore cross-cultural differences in attachment-related emotion processing.

Second, the use of static facial stimuli potentially reduces the ecological validity of our results. Real-world emotion perception often involves dynamic facial expressions embedded in rich social contexts [73]. Therefore, future research might benefit from adopting more complex experimental tasks, for example using social scenes rather than cropped faces without any social context [48]. Incorporating dynamic stimuli or multimodal approaches, such as combining eye-tracking with event-related potentials (ERP), could provide a more comprehensive understanding of attentional processes [49].

Third, eye movement indices only provided indirect evidence for possibly altered attentional processes related to anxiety or attachment insecurity [74]. Furthermore, fixation duration was the sole eye-tracking metric analyzed as a potential indicator of underlying cognitive mechanisms during the facial emotion recognition and face recognition tasks. While fixation duration offers valuable insights into cognitive effort, additional metrics such as pupil dilation or saccade patterns could enhance our understanding of attentional and memory mechanisms [50,55]. Future research might also benefit from implementing paradigms with longer viewing times, allowing longer or more fixations, which in turn might result in higher variance between participants (that might make the effect of individual attachment styles on fixation duration more prominent).

Lastly, the study’s focus on two basic emotions—happiness and sadness—may not fully capture the complexity of socio-emotional interactions. Future research should expand to include a broader range of emotions, such as fear, anger, or disgust, to explore their unique associations with attachment and anxiety.

Despite these limitations, our findings hold practical implications for both research and applied settings. The observed associations between attachment insecurity, anxiety, and emotion perception underscore the importance of considering individual differences in clinical interventions targeting emotion regulation. For example, therapies focusing on attachment security could mitigate attentional biases and improve socio-emotional functioning. Furthermore, the nuanced role of anxiety traits in modulating visual attention highlights the need for tailored interventions addressing specific anxiety dimensions.

In conclusion, this study provides preliminary evidence for the influence of individual attachment orientations and anxiety traits on visual attention during emotion perception and recognition tasks. While further research is needed to validate and extend these findings, our results offer a foundation for understanding how individual differences shape socio-emotional processing and highlight avenues for future exploration.

## 5. Conclusions

This study employed eye-tracking technology to explore how adult attachment orientations and anxiety traits influence facial emotion perception and face memory. By integrating the two-dimensional model of attachment and incorporating anxiety as a covariate, the research revealed that insecure attachment styles (both anxiety and avoidance dimensions) are associated with prolonged fixation durations on emotional faces, highlighting their role in attentional allocation to socio-emotional stimuli. Conversely, both trait and state anxiety were found to negatively correlate with fixation durations, underscoring the complex relationship between anxiety characteristics and attentional processes.

The methodology—which included two computerized tests, comprising an emotion decision and a face recognition task, channeled into two separate multilevel models—allowed for a nuanced investigation of how individual differences shape both perception and memory mechanisms. While the emotion decision task provided robust evidence for attachment-related differences in emotion-specific attentional patterns, the recognition task underscored the significant influence of trait anxiety on visual attention, albeit without clear effects from attachment dimensions.

These findings are important as they enhance our understanding of how attachment and anxiety modulate socio-emotional processing, with potential implications for clinical practices aimed at improving emotion regulation and interpersonal functioning. Despite its limitations, this study provides a foundation for future research to expand upon, particularly by incorporating dynamic stimuli, broader populations, and additional eye-tracking metrics. In doing so, this line of research holds promise for uncovering more comprehensive insights into the interplay between attachment, anxiety, and emotion processing.

## Figures and Tables

**Table 1:** Basic demographic and questionnaire measures of the study sample. Percentiles (25th, 50th, 75th) were used to determine reference values for the dependent variable estimates in LSmeans analyses.

N (No. of Male)		50 (26)				
Handedness		49 Right-Handed ^a^ (Based on EHI ^b^ Scores)				
Questionnaire Measures ^c^		Age	STAI-T	STAI-S	ECR_Avoidance	ECR_Anxiety
Mean		24.40	42.62	31.94	41.62	48.78
Median		22.50	42.00	32.00	41.50	49.50
Std. Deviation		4.84	7.709	6.268	17.467	19.153
Percentiles	25th	20.75	37.00	27.75	30.50	34.00
	50th	22.50	42.00	32.00	41.50	49.50
	75th	27.25	48.00	35.00	51.75	64.00

^a^ 1 participant did not complete the handedness assessment. ^b^ EHI = Edinburgh Handedness Inventory. ^c^ Age is also depicted here since it was also used as a “fixed effect” variable in data analysis.

**Table 2:** Summary of Model 1 results. The table shows differences in fixation duration measured during the Emotion Decision task, assessed with a three-level hierarchical model: fixation ranks (1st–3rd) nested within face regions (AoI-s: left eye, right eye, nose, mouth), nested within stimulus type (sad, happy, neutral).

Summary of Model 1 Results (Emotion Decision)		
Random Effects (Covariance Parameter Estimates)		*p*-Value
Fixation Rank		*p* = 0.039 (3.2% of variance)
AoI-s (Face Regions)		*p* = 0.045 (4.8% of variance)
Stimulus Type		*p* = 0.136 (12.1% of variance)
**Fixed Effects 1**	**F-value**	***p*-value**
STAI-T (Trait Anxiety)	F = 10.69	*p* < 0.01
STAI-S (State Anxiety)	F = 4.99	*p* = 0.026
ECR Avoidance	F = 5.69	*p* = 0.017
ECR Anxiety	F = 3.13	*p* = 0.077 *(tendency)*
Age	F = 0.79	*p* = 0.374
RT (“Sad” responses)	F = 1.24	*p* = 0.266
Accuracy (“Sad” responses)	F = 1.93	*p* = 0.165
RT (“Happy” responses)	F = 1.27	*p* = 0.259
Accuracy (“Happy” responses)	F = 0.43	*p* = 0.51
**Updated model *(after removal of non-significant fixed effects)***		
**Fixed Effects 2**	**F-value**	***p*-value**
STAI-T (Trait Anxiety)	F = 13.17	*p* < 0.001
STAI-S (State Anxiety)	F = 7.02	*p* < 0.01
ECR Avoidance	F = 3.11	*p* = 0.078 *(tendency)*
ECR Anxiety	F = 4.46	*p* = 0.035
**Main Effects**	**F-value**	***p*-value**
Stimulus Type	F = 135.94	*p* < 0.0001
AoI-s (Face Regions)	F = 12.09	*p* < 0.0001
Fixation Rank	F = 3.14	*p* = 0.0434
**Interactions**	**F-value**	***p*-value**
Stimulus Type × AoI	F = 9.93	*p* < 0.0001
Stimulus Type × Fixation Rank	F = 40.23	*p* < 0.0001
AoI × Fixation Rank	F = 8.18	*p* < 0.0001
Stimulus Type × AoI × Fixation Rank	F = 6.69	*p* < 0.0001

## Data Availability

The data that support the findings of this study are available from the corresponding author [KTS], upon reasonable request.

## Supplementary Materials

The supporting information for detailed LSmeans analyses can be downloaded at: https://www.mdpi.com/article/10.3390/jimaging11020060/s1.

## Abbreviations

The following abbreviations are used in this manuscript:

AoI	Area of Interest
ASD	Autism Spectrum Disorder
BDI-13	Beck Depression Inventory (13-item)
BPD	Borderline Personality Disorder
ECR	Experiences in Close Relationships questionnaire
ERP	Event-Related Potentials
RaFD	Radboud Faces Database
SAD	Social Anxiety Disorder
STAI	State-Trait Anxiety Inventory
