# Supplementary material for: Investigating Eye Movements to Examine Attachment-Related Differences in Facial Emotion Perception and Face Memory
Source: J Imaging. 2025 Feb 16;11(2):60. doi: 10.3390/jimaging11020060 (PMC11856241; doi:10.3390/jimaging11020060)
Supplement: Supplementary file 1 [file jimaging-11-00060-s001.zip › jimaging-3413554-supplementary.pdf]

Supplementary material

Section S1: Model 1 – Emotion Decision (Detailed results)

Model 1 assessed fixation duration within a three-level hierarchical structure: fixation rank (1st-3rd) nested within face regions (AoI-s: left eye, right eye, nose, mouth), which were nested within stimulus type (sad, happy, neutral). Fixation duration was the dependent variable, with accuracy, reaction time (RT), age, and questionnaire scores as fixed effects.

Based on the Covariance Parameter Estimates of the model, the random intercepts of fixation rank ($p = 0.039$), and AoI-s ($p = 0.045$) significantly explained 3.2% and 4.8% of the variance in fixation duration, respectively. However, even though stimulus type would have explained 12.1% of variance in fixation duration, it did not reach significance ($p = 0.136$), after accounting for the variance explained by fixation rank and AoI-s.

Analysis of fixed effects also revealed several significant results: scores on the STAI-T ($F = 10.69$, $p < 0.01$), STAI-S ($F = 4.99$, $p = 0.026$), and the avoidance dimension of ECR ($F = 5.69$, $p = 0.017$) provided an estimate of fixation duration that differed significantly from 0 (no effect), while the anxiety dimension of ECR showed a tendency for significance ($F = 3.13$, $p = 0.077$). All other fixed effects (age: $p = 0.374$, sad RT: $p = 0.266$, sad accuracy: $p = 0.165$, happy RT: $p = 0.259$, and happy accuracy: $p = 0.51$) did not reach significance, therefore these variables were not included in subsequent analyses.

After removing non-significant fixed effects, the model was updated to incorporate interactions of classification variables (fixation rank, AoI-s, and stimulus type), as well as calculating LSmeans as post-hoc comparisons between each level of the clustered data at different levels of fixed effects (i.e., questionnaire scores). As shown previously, the attachment avoidance and anxiety dimensions measured by the ECR questionnaire should not be treated independently [S1], therefore attachment anxiety, which only showed a tendency for significance in the baseline model was kept as a covariate throughout subsequent LSmeans analyses. Removal of all other non-significant covariates improved the model fit and resulted in different estimates for the remaining fixed effects: scores on the STAI-T ($F = 13.17$, $p < 0.001$) and STAI-S ($F = 7.02$, $p < 0.01$) remained strongly significant, while the avoidance dimension of ECR now showed only a tendency for significance ($F = 3.11$, $p = 0.078$). The anxiety dimension of ECR ($F = 4.46$, $p = 0.035$) also showed a significant effect, after removing all other non-significant variables from the model.

The updated model also revealed that stimulus type ($F = 135.94$, $p < 0.0001$), AoI-s ($F = 12.09$, $p < 0.0001$) and fixation rank ($F = 3.14$, $p = 0.0434$) all had significant main effects on fixation duration. Interactions

between different levels of the model were also significant: stimulus type × AoI ($F = 9.93$, $p < 0.0001$), stimulus type × fixation rank ($F = 40.23$, $p < 0.0001$), AoI × fixation rank ($F = 8.18$, $p < 0.0001$), and stimulus type × AoI × fixation rank ($F = 6.69$, $p < 0.0001$); these results revealed that fixation duration differed significantly at different values of hierarchical variables.

Post-hoc analysis of Differences of Least Squares Means revealed significant differences between all three types of emotional expressions (i.e., stimulus type): fixations were longest for sad faces ($t = 30.82$, $p < 0.0001$), shorter for happy ($t = 18.33$, $p < 0.0001$), and shortest for neutral faces ($t = 7.67$, $p < 0.0001$).Furthermore, fixation lengths differed significantly between sad and happy ($t = 8.84$, $p < 0.0001$), sad and neutral ($t = 16.37$, $p < 0.0001$), and happy and neutral faces as well ($t = 7.53$, $p < 0.0001$). At the level of AoI-s, only the left eye region differed significantly from all other areas (right eye: $t = 3.43$, $p < 0.01$; nose: $t = 4.11$, $p < 0.001$; mouth: $t = 5.82$, $p < 0.0001$). Fixation ranks (1-3.) did not differ significantly from each other (1-2: $p = 0.99$; 1-3: $p = 0.084$; 2-3: $p = 0.074$). Given the lack of differences between fixation ranks, these were not included in any further analyses.

Differences of LSmeans of the stimulus type × AoI interaction were further analysed in the context of fixed effect covariates (ECR avoidance, ECR anxiety, STAI-T, and STAI-S scores). As seen in Table 1, percentile scores were calculated for all questionnaires to determine reference values for the calculated estimates of the dependent variable. The model was used to estimate fixation duration according to scores (25th, 50th and 75th percentile) on a given questionnaire, by calculating a linear estimate based on actual values of participants (considering the covariates as well).

Section S2: Model 1 - Post-hoc analysis: Interaction of *stimulus type* and *AoI-s* at different levels of attachment avoidance (ECR Avoidance scores)

The interaction of stimulus type and AoI-s revealed specific patterns of fixations for different emotions, in the context of attachment avoidance: at the 25th percentile (30.5 points) all AoI-s differed significantly for sad faces (left eye: $t = 16.92$; right eye: $t = 17.59$; nose: $t = 15.45$; mouth: $t = 9.56$; all p-s < 0.0001). AoI-s for happy faces also revealed significant differences (left eye: $t = 8.79$; right eye: $t = 7.47$; nose: $t = 8.17$; mouth: $t = 10.41$; all p-s < 0.0001). However, for neutral faces, only the left eye AoI showed a significant effect ($t = 9.40$; $p < 0.0001$), all other AoI-s were not significant (right eye: $p = 0.08$; nose: $p = 0.12$; mouth: $p = 0.28$).

Results were similar for the 50th percentile (41.5 points) on the ECR avoidance scale: all AoI-s differed significantly for sad faces (left eye: $t = 17.58$; right eye: $t = 18.26$; nose: $t = 16.08$; mouth: $t = 10.08$; all p-s < 0.0001). AoI-s for happy faces also revealed significant differences (left eye: $t = 9.30$; right eye: $t = 7.95$; nose: $t = 8.67$; mouth: $t = 10.94$; all p-s < 0.0001). For neutral faces, the left eye ($t = 9.92$; $p < 0.0001$) and

right eye (t = 2.15; p = 0.032) both showed a significant effect, other AoI-s were not significant (nose (*tendency*): p = 0.0552; mouth: p = 0.15).

At the 75th percentile (51.75 points) again all AoI-s differed significantly for sad faces (left eye: t = 17.62; right eye: t = 18.29; nose: t = 16.14; mouth: t = 10.24; all p-s < 0.0001). AoI-s for happy faces were also significant (left eye: t = 9.47; right eye: t = 8.14; nose: t = 8.85; mouth: t = 11.09; all p-s < 0.0001). For neutral faces, the left eye (t = 10.08; p < 0.0001) and right eye (t = 2.43; p = 0.015), as well as the nose area (t = 2.20; p = 0.028) showed a significant effect, while the mouth AoI still remained non-significant (p = 0.085).

Section S3: Model 1 - Post-hoc analysis: Interaction of *stimulus type* and *AoI-s* at different levels of attachment anxiety (ECR anxiety scores)

The interaction between stimulus type and AoI-s showed specific patterns of fixation durations for different emotions, in the context of attachment anxiety: at the 25th percentile (34 points) all AoI-s differed significantly for sad faces (left eye: t = 16.41; right eye: t = 17.07; nose: t = 14.97; mouth: t = 9.18; all p-s < 0.0001). AoI-s for happy faces also revealed significant differences (left eye: t = 8.43; right eye: t = 7.12; nose: t = 7.81; mouth: t = 10.01; all p-s < 0.0001). However, for neutral faces, only the left eye AoI showed a significant effect (t = 9.02; p < 0.0001), all other AoI-s were not significant (right eye: p = 0.13; nose: p = 0.19; mouth: p = 0.40).

Results were similar for the 50th percentile (49.5 points) on the ECR anxiety scale: all AoI-s differed significantly for sad faces (left eye: t = 17.61; right eye: t = 18.29; nose: t = 16.11; mouth: t = 10.11; all p-s < 0.0001). AoI-s for happy faces also differed significantly (left eye: t = 9.33; right eye: t = 7.98; nose: t = 8.70; mouth: t = 10.97; all p-s < 0.0001). For neutral faces, the left eye (t = 9.95; p < 0.0001) and right eye (t = 2.18; p = 0.029) both showed a significant effect, other AoI-s were not significant (nose (*tendency*): p = 0.0513; mouth: p = 0.14).

At the 75th percentile (64 points) again all AoI-s differed significantly for sad faces (left eye: t = 17.50; right eye: t = 18.16; nose: t = 16.06; mouth: t = 10.28; all p-s < 0.0001). AoI-s for happy faces were also significant (left eye: t = 9.53; right eye: t = 8.23; nose: t = 8.92; mouth: t = 11.11; all p-s < 0.0001). For neutral faces, the left eye (t = 10.13; p < 0.0001) and right eye (t = 2.64; p < 0.01), as well as the nose area (t = 2.42; p = 0.016) showed a significant effect, while the mouth AoI showed a tendency for significance (p = 0.0511).

Section S4: Model 1 - Post-hoc analysis: Interaction of *stimulus type* and *AoI-s* at different levels of trait anxiety (STAI-T scores)

The interaction between stimulus type and AoI-s revealed specific patterns of fixations for different emotions, in the context of trait anxiety: at the 25th percentile (37 points) all AoI-s differed significantly for sad faces (left eye: $t = 17.92$; right eye: $t = 18.58$; nose: $t = 16.48$; mouth: $t = 10.68$; all p-s < 0.0001). AoI-s for happy faces also differed significantly (left eye: $t = 9.93$; right eye: $t = 8.62$; nose: $t = 9.32$; mouth: $t = 11.51$; all p-s < 0.0001). For neutral faces, all AoI-s showed a significant effect as well (left eye: $t = 10.53$, $p < 0.0001$; right eye: $t = 3.02$, $p < 0.01$; nose: $t = 2.80$, $p < 0.01$; mouth: $t = 2.33$, $p = 0.02$).

At the 50th percentile (42 points) of the STAI-T scale, all AoI-s differed significantly for sad faces (left eye: $t = 17.69$; right eye: $t = 18.37$; nose: $t = 16.19$; mouth: $t = 10.19$; all p-s < 0.0001). AoI-s for happy faces also differed significantly (left eye: $t = 9.41$; right eye: $t = 8.06$; nose: $t = 8.78$; mouth: $t = 11.05$; all p-s < 0.0001). For neutral faces, the left eye ($t = 10.03$; $p < 0.0001$) and right eye ($t = 2.26$; $p = 0.02$), as well as the nose area ($t = 2.03$; $p = 0.04$) showed a significant effect, while the mouth AoI was not significant ($p = 0.12$).

At the 75th percentile (48 points) again all AoI-s differed significantly for sad faces (left eye: $t = 16.13$; right eye: $t = 16.78$; nose: $t = 14.67$; mouth: $t = 8.86$; all p-s < 0.0001). AoI-s for happy faces were also significant (left eye: $t = 8.11$; right eye: $t = 6.80$; nose: $t = 7.49$; mouth: $t = 9.70$; all p-s < 0.0001). However, for neutral faces, only the left eye AoI showed a significant effect ($t = 8.71$; $p < 0.0001$), all other AoI-s were not significant (right eye: $p = 0.24$; nose: $p = 0.34$; mouth: $p = 0.63$).

Section S5: Model 1 - Post-hoc analysis: Interaction of *stimulus type* and *AoI-s* at different levels of state anxiety (STAI-S scores)

The interaction between stimulus type and AoI-s showed specific patterns of fixation durations for different emotions, in the context of state anxiety: at the 25th percentile (27.75 points) all AoI-s differed significantly for sad faces (left eye: $t = 17.77$; right eye: $t = 18.43$; nose: $t = 16.29$; mouth: $t = 10.41$; all p-s < 0.0001). AoI-s for happy faces also differed significantly (left eye: $t = 9.64$; right eye: $t = 8.31$; nose: $t = 9.02$; mouth: $t = 11.25$; all p-s < 0.0001). For neutral faces, the left eye ($t = 10.25$; $p < 0.0001$) and right eye ($t = 2.62$; $p < 0.01$), as well as the nose area ($t = 2.40$; $p = 0.016$) showed a significant effect, while the mouth AoI showed a tendency for significance ($p = 0.055$).

At the 50th percentile (32 points) of the STAI-S, again all AoI-s differed significantly for sad faces (left eye: $t = 17.58$; right eye: $t = 18.26$; nose: $t = 16.08$; mouth: $t = 10.08$; all p-s < 0.0001), as well as happy faces (left eye: $t = 9.30$; right eye: $t = 7.94$; nose: $t = 8.67$; mouth: $t = 10.94$; all p-s < 0.0001). For neutral faces,

the left eye ($t = 9.92$; $p < 0.0001$) and right eye ($t = 2.14$; $p = 0.032$) both showed a significant effect, other AoI-s were not significant (nose (*tendency*): $p = 0.056$; mouth: $p = 0.15$).

At the 75th percentile (48 points) again all AoI-s differed significantly for sad faces (left eye: $t = 17.03$; right eye: $t = 17.70$; nose: $t = 15.55$; mouth: $t = 9.61$; all p-s < 0.0001). AoI-s for happy faces were also significant (left eye: $t = 8.84$; right eye: $t = 7.49$; nose: $t = 8.21$; mouth: $t = 10.46$; all p-s < 0.0001). For neutral faces, only the left eye AoI showed a significant effect ($t = 9.45$; $p < 0.0001$), all other AoI-s were not significant (right eye: $p = 0.08$; nose: $p = 0.13$; mouth: $p = 0.30$).

Section S6: Model 2 - Post-hoc analysis: Interaction of AoI-s and fixation ranks at different levels of trait anxiety

The interaction between AoI-s and fixation ranks revealed specific patterns of fixations for different face regions, in the context of trait anxiety: at the 25th percentile (37 points) all fixations differed significantly for the left eye (1st fixation: t = 19.29; 2nd: t = 13.55; 3rd: t = 11.45; 4th: t = 14.03; 5th: t = 11.71; all p-s < 0.0001). Fixations on the right eye also differed significantly (1st fixation: t = 6.27; 2nd: t = 7.94; 3rd: t = 13.82; 4th: t = 10.99, 5th: t = 10.73; all p-s < 0.0001). All fixations showed a significant effect for the nose AoI as well (1st fixation: t = 4.75; 2nd: t = 7.11; 3rd: t = 7.93; 4th: t = 9.54, 5th: t = 5.73; all p-s < 0.0001). However, the first 2 fixations for the mouth area were not significant (1st: p = 0.68, 2nd: p = 0.06), all other fixations differed significantly (3rd: t = 3.49, p < 0.001; 4th: t = 4.95, p < 0.0001; 5th: t = 5.38, p < 0.0001).

At the 50th percentile (42 points) of the STAI-T scale, again all fixations differed significantly for the left eye (1st fixation: t = 19.21; 2nd: t = 13.39; 3rd: t = 11.27; 4th: t = 13.88; 5th: t = 11.53; all p-s < 0.0001). Fixations on the right eye also differed significantly (1st fixation: t = 6.02; 2nd: t = 7.71; 3rd: t = 13.67; 4th: t = 10.80, 5th: t = 10.54; all p-s < 0.0001). Fixation rank showed a significant effect for the nose AoI as well (1st fixation: t = 4.48; 2nd: t = 6.87; 3rd: t = 7.70; 4th: t = 9.33, 5th: t = 5.47; all p-s < 0.0001). The first 2 fixations for the mouth area were again not significant (1st: p = 0.93, 2nd: p = 0.12), all other fixations differed significantly (3rd: t = 3.20, p < 0.01; 4th: t = 4.68, p < 0.0001; 5th: t = 5.12, p < 0.0001).

At the 75th percentile (48 points) all fixations differed significantly for the left eye (1st fixation: t = 18.58; 2nd: t = 12.84; 3rd: t = 10.74; 4th: t = 13.31; 5th: t = 10.99; all p-s < 0.0001). Fixations to the right eye also differed significantly (1st fixation: t = 5.55; 2nd: t = 7.22; 3rd: t = 13.11; 4th: t = 10.27, 5th: t = 10.02; all p-s < 0.0001). All fixations showed a significant effect for the nose AoI as well (1st fixation: t = 4.03; 2nd: t = 6.39; 3rd: t = 7.21; 4th: t = 8.82, 5th: t = 5.00; all p-s < 0.0001). The first 2 fixations for the mouth area were again not significant (1st: p = 0.75, 2nd: p = 0.25), all other fixations differed significantly (3rd: t = 2.76, p < 0.01; 4th: t = 4.23, p < 0.0001; 5th: t = 4.66, p < 0.0001).

References

S1. Cameron, J. J., Finnegan, H., & Morry, M. M. (2012). Orthogonal dreams in an oblique world: A meta-analysis of the association between attachment anxiety and avoidance. Journal of Research in Personality, 46(5), 472-476. https://doi.org/10.1016/j.jrp.2012.05.001
